# Rhabdomyosarcoma of the head and neck in pediatric patients: a systematic review

**DOI:** 10.4317/medoral.25508

**Published:** 2022-09-29

**Authors:** Mariana P Rodríguez-Vargas, Francisco G Villanueva-Sánchez

**Affiliations:** 1 Dental Science, National School of Higher Studies León Unit, National Autonomous University of Mexico, Mexico; 2 Coordinator of the Oral and Maxillofacial Pathology Service, National School of Higher Studies León Unit, National Autonomous University of Mexico, Mexico

## Abstract

**Background:**

Rhabdomyosarcoma (RMS) is a soft tissue malignant tumor of mesenchymal cell origin, which usually shows variable differentiation of muscle cells. It is the most common solid sarcoma in children. The most usual site of occurrence are the head and neck regions. RMS presents a variety of histologic features, and so differential diagnosis with other small round cell tumors is needed. Hence, it has been very useful to the field to undertake additional immunohistochemical studies to determine the diagnosis and, on occasions, to assign subtype tumors.

**Material and Methods:**

A systematic review of three databases (Medline, Biological Science Collection and Health & Medical Collection) was carried out with the purpose of analyzing rhabdomyosarcoma cases reported in the literature, specifically with localization in the head and neck regions in children. This strategy allowed us to identify the main anatomical site of appearance, the subtype of RMS, average age, histologic characteristics and immunohistochemistry markers used in a usual and any additional way.

**Results:**

According to the selection criteria in this systematic review, twelve articles, and fourteen cases were identified that highlight that the histological diagnosis usually presents cellular heterogeneity. Therefore, immunohistochemistry is needed to confirm the diagnosis.

**Conclusions:**

Histologic characterization is not always sufficient for a conclusive diagnosis of RMS. Therefore, immunohistochemistry is helpful to determine the subtype and consequently, sometimes the behavior, treatment and prognosis. Additional markers may vary according to the institution and the need of particular cases.

** Key words:**Pediatric, rhabdomyosarcoma, head and neck, histologic, immunohistochemistry.

## Introduction

Rhabdomyosarcoma (RMS) is a soft tissue malignant tumor of mesenchymal cell origin with cellular diversity and variable differentiation of muscle cells. It represents approximately 5% of malignant neoplasms in children, being the most common sarcoma in pediatric age (1). More than 50% of RMS are diagnosed within the first decade of life. According to the World Health Organization classification, four main RMS subtypes can be identified: embryonal, alveolar, pleomorphic, and spindle/sclerosing RMS (2). The most common site of occurrence are the head and neck regions, followed by the genitourinary tract and the extremities (3).

It is reported in previous literature that RMS can present a variety in their histologic features: especially the embryonal and alveolar subtypes can show cellularity with appearance of small and round, primitive cells. Consequently differential diagnosis with other small round cell tumors is needed. In this regard, it is necessary to take advantage of techniques with immunohistochemical markers in order to obtain an accurate and specific diagnosis (4).

The aim of this study was to review the cases reported in the literature of rhabdomyosarcomas with localization in the head and neck regions in the pediatric age. This review allows to describe the main anatomical structures involved, the RMS subtypes, and the average age of presentation. Furthermore, in this study, we can highlight the importance of the histological features description found in the included cases, and in the most recent classification, as well as the immunohistochemical analysis, in order to obtain an accurate diagnosis, which will have repercussion and improvement in clinical practice.

## Material and Methods

- Search strategy

An advanced search was performed in the MEDLINE database using the words “pediatric” AND “rhabdomyosarcoma” AND “head and neck.” This search was applied to any section of the document, limited to case reports, clinical studies, and clinical trials, and published between 2015 and 2022. The result was 34 articles, of which 10 were excluded due to title and abstract: 4 of them dealt with cases in children, but not in the region of interest; 5 more dealt with cases in adult patients, and two more were review articles. Finally, according to inclusion criteria, we selected 10 articles.

A new advanced search was introduced in the HEALTH & MEDICAL COLLECTION database. The same terms were used in the Medical Subject Headings (MeSH) “pediatric,” “rhabdomyosarcoma,” and “head and neck”. These three terms were combined with the Boolean command AND, published between 2015 and 2022, in the English language. A result of 54 articles was obtained: 31 articles were ruled out by the title; 1 was duplicated; 9 of them dealt with cases out of the anatomical region of interest; 11 more dealt with cases in adult patients, and 2 remaining were included by title and abstract.

An advanced search of the BIOLOGICAL SCIENCE COLLECTION database was conducted to find documents of the type case study and article, published between 2015 and 2022, in the English language, using the words “pediatric” AND “rhabdomyosarcoma” AND “head and neck.” 44 articles were found, of which 18 were excluded due to title, 11 were duplicated, 6 articles dealt with cases out of the anatomical region of interest, 8 more dealt with cases in adult patients, and one more was a review article. We eliminated from the analysis all of the articles found in this database for the reasons mentioned above, so only 12 articles from the first two databases form part of this systematic review.

- Inclusion criteria

Overall, the following inclusion criteria were used to select the articles for the systematic review:

1) Case reports, clinical studies, and clinical trials type of articles.

2) Cases with histological confirmation of the diagnosis of rhabdomyosarcoma.

3) Immunohistochemical and/or molecular analysis.

4) Localization of the primary tumor in the head and neck area.

5) Presentation in pediatric age (children from birth to adolescence, up to eighteen years of age).

6) Articles published in the period from 2015 to 2022.

- Exclusion criteria

The following deviations from the inclusion criteria served as a reason to exclude articles from the review:

1) Pure literature review articles without original research.

2) Articles of cases in adults, even when the location of the tumor is in the region of interest.

3) Cases were excluded where there is a diagnosis of RMS in children, but the location of the neoplasm is in a part of the body other than the head or neck.

4) Articles with no mention of histological or immunohistochemical diagnostic confirmation.

## Results

**Figure 1:**
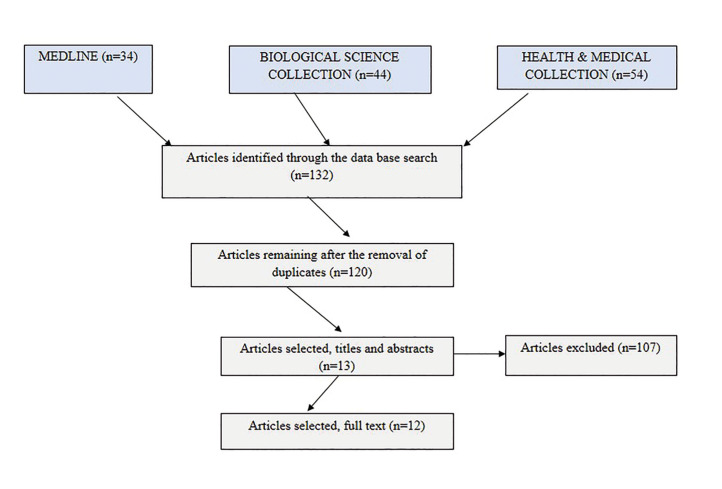
PRISMA flow chart of the literature and article selection.

According to the selection criteria, twelve articles were included in this systematic review (Fig. 1) (5) represents the PRISMA flow chart of the literature and article selection. We analyzed a total of fourteen RMS cases described in the twelve articles reviewed.

The included articles describe case reports of rhabdomyosarcoma in the head and neck region in patients of pediatric age. In these articles, the authors report the following anatomical locations of occurrence of primary tumors: 4 in paraspinal muscles of the neck, 3 in regions of the temporal bone, 2 in paranasal sinuses (one of them involving right sphenoid and ethmoidal sinuses, with extension to the right medial orbital wall; the second of them, with a wide invasion, was occupying the left sinonasal area, with intracranial extension, involving the cavernous sinus, bifrontal extradural space, and left temporal lobe; the mass also extended to both orbits, the left nasopharynx, and the oropharynx part), 1 in the maxilla, 1 in the mandible, 1 in the buccal mucosa, 1 in the conjunctiva, and 1 in the left dorsal aspect of the tongue (Table 1).

In terms of tumor subtype, the following are found: 2 alveolar; 3 spindle cell/sclerosing, 6 embryonal RMS, and finally 3 cases described as a homogenous type of well-differentiated RMS, characterized by specific translocations of the SRF (Serum Response Factor) gene.

Table 2 summarizes this information for each case reported in the twelve articles, (6-17) taking into account the histological description with hematoxylin and eosin (H&E) staining and the WHO classification. The age range of patients at diagnosis is from six weeks to thirteen years. Six of the cases reported in the sample occurred in girls and eight in boys.

The dimensions of the tumors at diagnosis are shown in Table 1 (6-17). The smallest tumor measured 1.2 cm and the largest tumor was 6.5 cm in diameter.

For the histologic description, each case presents some particularity, especially according to the location. However, they share some common characteristics. For example, in the embryonal subtype, small round and blue cells with hyperchromatic nuclei and eosinophilic cytoplasm were observed. In the case of the alveolar type, the cells are separated by fibrovascular septae. And in the spindle cell subtype, a myxoid stroma contained round and spindled cells with hyperchromatic nuclei and scanty cytoplasm.

Particularly, in the paper by Karanian *et al*. (14) they report three cases of rhabdomyosarcoma in paraspinal neck muscles and describe them as a homogeneous type of well-differentiated RMS with translocations of the SRF gene. According to the report just mentioned, the reason the cases are not classified as alveolar or spindle cell/sclerosing types is that this histologic pattern described as well-differentiated is not usual in those types of RMS. In the case of embryonal RMS type, in fact, it is commonly composed of mesenchymal cells at different stages of myogenesis, with rhabdomyoblasts, and mixed with less differentiated cells. That could well describe those three cases. However, within the molecular analysis, they found new fusion genes, SRF-FOXO1 and SRF-NCOA1, and these anomalies cast doubt on the classification of embryonal RMS. Thus, the authors report that histological and genomic analyses suggest that the SRF-fused RMS may be a new specific subtype of skeletal muscle tumor.

Regarding the immunohistochemical markers used, summatively the authors of the sample of articles report a strong positivity for vimentin, desmin, myoglobin, and muscle specific actin in the alveolar type (Shruta *et al*., 2015). The authors found cytoplasmic positivity for desmin and nuclei positivity for myogenin, in embryonal rhabdomyosarcoma, and also in one case positivity for MyoD1 (Peters S., Silva J., Morales L., Beghdad M., Bhandarkar A.,and Shahidatul-Adha M.).

**Table 1:**
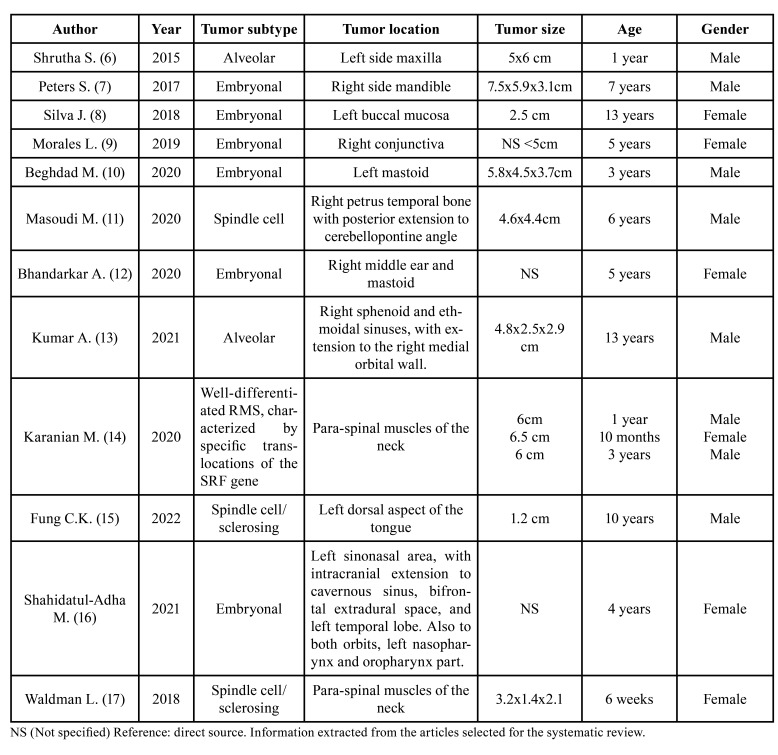
Summary of relevant data extracted from the articles included in the review.

**Table 2:**
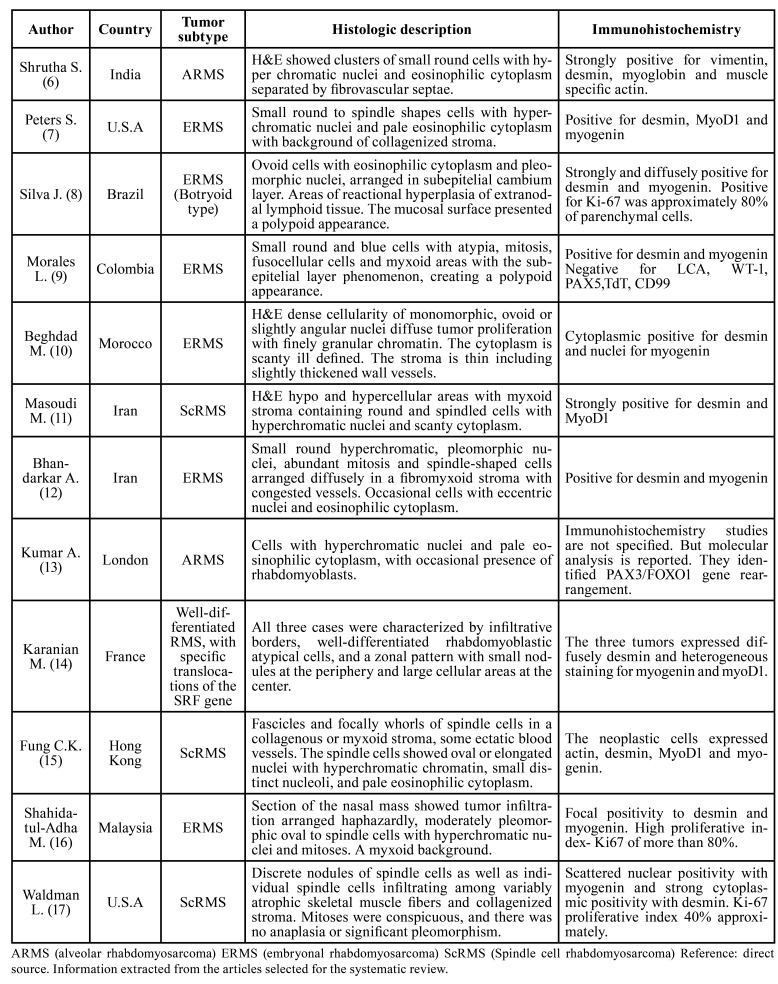
Histologic features reported in the articles included in this review.

And finally, in the spindle cell subtype, strong positivity was observed for desmin and MyoD1 (Masoudi M. *et al*.), as well as positivity for myogenin in two other cases (Fung C.K., and Waldman L.). The cases described as well-differentiated RMS, with specific translocations of the SRF gene, diffusely expressed desmin and heterogeneous staining for myogenin and myoD1 (Karanian M. *et al*.). It should be noted that the antibody mostly observed was myogenin. Therefore, we cannot affirm, in the sample of these cases, that an immunomarker is specific or more commonly positive in a certain subtype of rhabdomyosarcoma.

## Discussion

This systematic review of literature allows us now to closely examine the following RMS characteristics: anatomical location, histologic subtypes and characteristics, immunohistochemical analyses, and clinicopathological and molecular features.

Anatomical location

Rhabdomyosarcoma (RMS) is the most common soft tissue sarcoma of pediatric onset. According to Iatrou *et al*. (18) report, head and neck RMS represent approximately 35% of the conjunctive tissue malignancies in children under nine years of age. The most common ones for children and adolescents are those of the extremities, paratesticular, and genitourinary tract.

The anatomical location in the head and neck region can be either parameningeal or non-parameningeal. The former are located either in the nasopharynx, nasal cavities, paranasal sinuses, infratemporal and pterygopalatine fossae, and middle ear. The non-parameningeal regions may include any other sites within the head and neck (19). In this review, primary tumors were most frequently found in paraspinal muscles of the neck, followed by temporal bone sites. The anatomical location is relevant, since it influences the prognosis and choice and course of treatment. In general, parameningeal RMS commonly presents with advanced disease and poor prognosis.

Histologic subtypes and characteristics

The main two histologic subtypes are embryonal and alveolar. The embryonal subtype represents the most common RMS, accounting for approximately 70% of cases reported; and typically appears in younger children. And the alveolar subtype tends to occur in older children and adolescents. Also, it is reported that the region of the head and neck used to be more affected by embryonal RMS as well, but less common by spindle cell RMS (20). In this revision, the main rhabdomyosarcoma subtype diagnosed in the reported cases was the embryonal type, followed by spindle cell/sclerosing RMS.

With respect to histological characteristics, and as it is summarized in Table 2 (6-17); according to the subtype, they share the main microscopic description. Microscopic findings with H&E staining described in a detailed manner in the 5th edition of the World Health Organization (WHO) soft tissue and bone tumors classification are as follows: For the embryonal subtype, typical ERMS are composed of variable differentiated rhabdomyoblasts within loose, myxoid mesenchyme, with alternating areas of dense and loose cellularity. The relative amount of myxoid matrix and spindled cells is highly variable. Occasional poorly differentiated ERMSs consist largely of primitive round cells. ARMS is highly cellular and composed of primitive round cells with scant cytoplasm and hyperchromatic nuclei. Tumor cells are arranged in nests separated by fibrovascular septa, which frequently exhibit loss of cellular cohesion in the center, conferring a pattern of irregular alveolar spaces. The spindled neoplastic cells have pale eosinophilic cytoplasm and blunted, ovoid fusiform, centrally located nuclei with small inconspicuous nucleoli. Mitotic Figures, nuclear atypia, and hyperchromatic nuclei are often present (21).

Within the WHO classification; since 2013, and later in the 2020 version, rhabdomyosarcomas were classified as follows according to their histological patterns: embryonal rhabdomyosarcoma, with botryoid and anaplastic variants; alveolar rhabdomyosarcoma, including a solid variant; spindle cell/sclerosing rhabdomyosarcoma and pleomorphic rhabdomyosarcoma, the latter occurs mostly in adults.

Immunohistochemical analyses

On the other hand, after histopathological diagnosis, immunohistochemical studies are a very useful tool. Alveolar rhabdomyosarcoma is recognized by staining strongly for myogenin (>50% of tumor cell nuclei), either in its classical or solid variants. Embryonal rhabdomyosarcomas use to be heterogeneous in their histological patterns; as well as the variable myogenin staining that they could show (ranging from 10% to 90% of tumor cells nuclei). Spindle cell/sclerosing rhabdomyosarcoma, generally has weak to absent myogenin staining, but strong nuclear MYOD1 staining (22). In the cases of this review there was observed strongly positive for vimentin, desmin, myoglobin, and muscle specific actin in the alveolar case. Cytoplasmic positive for desmin and nuclei positive for myogenin in embryonal rhabdomyosarcoma. And strongly positive for desmin and MyoD1 in the spindle cell rhabdomyosarcoma case. In the consensus reported by Rudzinsky *et al*. the main markers in the three groups are myogenin, desmin, and MyoD, which are expected to be positive.

Clinicopathological and molecular features

Interestingly, in addition to having already been considered as a separate subtype in the 2013 WHO classification, spindle cell/sclerosing rhabdomyosarcoma, in the 2020 update, there have been described three subgroups taking into account clinicopathological and molecular features. So now there can be identified tumors with mutations in MYOD1, mostly seen in adolescents and young adult patients. Then, there are tumors with gene fusions which includes VGLL2, NCOA2 or CITED2 that mainly is found in pediatric patients and it has been related to a better prognosis. And the last one, tumors with no one of these alterations. And also recent characterization of spindle cell/sclerosing rhabdomyosarcoma with TFCP2 rearrangement or MEIS-NCOA2 fusion that have been related to bone tumors (23).

In this review we found three cases with a diagnosis of spindle cell/sclerosing rhabdomyosarcoma, one of them does not report molecular findings. The case described by Fung *et al*. (15) report a DCTN1::ALK fusion, joining exon 26 of the DCTN1 gene and exon 20 of the ALK gene. And they did not find a mutation in the MYOD1 L122 region. Waldman *et al*. (17) report the presence of VGLL2 gene rearrangement, and no abnormalities in the NCOA2 gene.

Agaram *et al*. (24) reported the identification of recurrent mutations in MYOD, related to an aggressive tumoral behavior. Alaggio and coworkers (25) identified VGLL-2-related fusions in spindle cell/sclerosing rhabdomyosarcoma in children. And also Mosquera *et al*. (26) reported a gene rearrangement in spindle cell rhabdomyosarcoma in pediatric patients. To mention a few, and which are currently taken into consideration, in specific cases as part of the molecular diagnosis, and as an important point in the therapy to be followed.

Immunohistochemistry markers used by the main study groups in the field

In order to enrich the present review, we include information reported by Rudzinski *et al*. (22) in their publication about a consensus of diagnostic criteria compiled from the leading study groups in the field: Children´s Oncology Group (COG), European Paediatric Soft Tissue Sarcoma Study Group (EpSSG), and the Cooperative Weichteilsarkom Studiengruppe (CWS). These three groups use the 2020 WHO RMS histological classification. Also, as used in the cases reported in this review, the following immunohistochemical markers are employed for diagnosis confirmation: myogenin, desmin, and MyoD1. All three groups report strong positivity for myogenin >50% of ARMS nuclei. According to them, ARMS also require FISH analysis for detection of the PAX3/PAX7-FOXO1 fusion gene.

All groups employ additional immunohistochemical analysis in order to determine the subtype, and they present slight differences between tests. According to their logarithms and according to histological patterns, in the case of embryonal type, variable myogenin staining may be present, ranging from 10% to 90% of neoplastic cell nuclei. For ERMS, COG includes nuclear HMGA2 positivity and relatively weak staining for AP2beta. In the same subtype, EpSSG includes HMGA2 and EGFR positivity and the absence of staining for AP2beta and P-Cadherin. And lastly, CWS uses positivity for EGFR and negative staining for P-Cadherin. COG for ARMS confirmation diagnosis uses and recognizes strong diffuse nuclear AP2beta staining, with weak to absent HMGA2 staining. EpSSG employs the same two last for ARMS, and additionally positivity for P-Cadherin and negative for EGFR. CWS, on the other hand, requires P-Cadherin positivity and negativity for EGFR. In the case of the spindle cell/sclerosing subtype the staining for myogenin use to be weak or even absent, but it shows strong nuclear MYOD1 staining (summarized in Table 3), information adapted from the article by Rudzinski E. *et al*. (22).

Although there are certain particularities as to how each group assigns the tumor subtype, there is a general consensus among pathologists as to what are considered minimal or standard clinical diagnostic criteria, addressing the criteria and characteristics described by the WHO. Consideration will only be necessary in those special cases where the histology is ambiguous (i.e. Karanian M. *et al*.) or a specific marker is suggested to influence the tumor behavior, prognosis, or plan of treatment. It should also be noted that it has been possible to identify an increasing variety of molecular anomalies in this type of sarcomas with the use of molecular techniques.

## Conclusions

Rhabdomyosarcomas (RMS) are soft tissue sarcomas, which more frequently occur in the pediatric age, and a large percentage within the first decade of life. A systematic review of relevant literature has been conducted, identifying and analyzing fourteen cases across twelve comparable scientific articles. About half of RMS cases are diagnosed in the head and neck region. The most frequent subtype is embryonal. In the histological diagnosis, they usually present cellular heterogeneity, distinctively the phenotype of cells with differentiation to skeletal muscle and primitive round and hyperchromatic cells. This sometimes makes an accurate diagnosis difficult. In most cases, as seen in the reviewed sample, histomorphologic analysis by hematoxylin and eosin staining with additional myogenin immunohistochemistry is sufficient to confirm the diagnosis and define the subtype. On occasions, other markers or molecular analysis will be necessary to recognize the histologic subtype and its potential incorporation into diagnostic and prognostic tests, as well as their possible influence on therapeutic options. We could highlight the importance of the histological features description, as well as the immunohistochemical and molecular analysis, in order to obtain an accurate diagnosis, which will have repercussion and improvement in clinical practice.

**Table 3:**
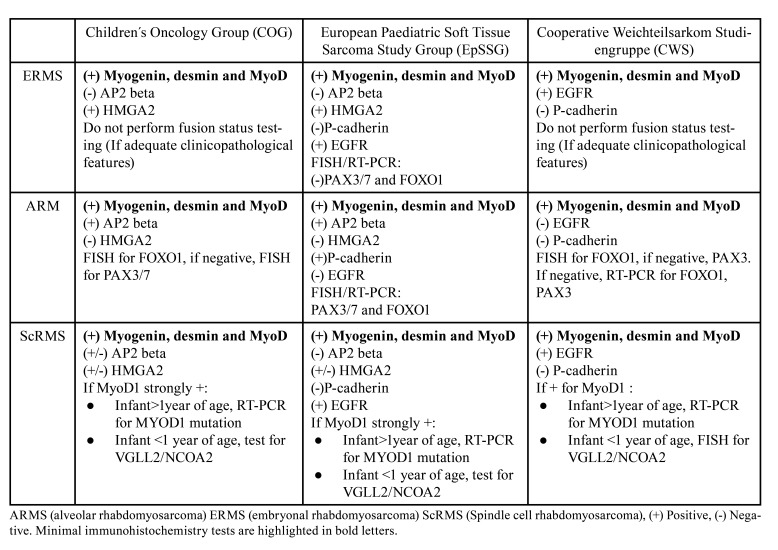
Diagnostic algorithm used by the three leading groups in the field of RMS diagnosis.

## References

[1] Leiner J, Le Loarer F (2020). The current landscape of rhabdomyosarcomas: an update. Virchows Arch.

[2] Bailey KA, Wexler LH (2020). Pediatric rhabdomyosarcoma with bone marrow metastasis. Pediatr Blood Cancer.

[3] Gartrell J, Pappo A (2020). Recent advances in understanding and managing pediatric rhabdomyosarcoma. F1000Res.

[4] Chen E, Ricciotti R, Futran N, Oda D (2017). Head and Neck Rhabdomyosarcoma: Clinical and Pathologic Characterization of Seven Cases. Head and Neck Pathol.

[5] Moher D, Liberati A, Tetzlaff J, Altman DG (2010). PRISMA Group. Preferred reporting items for systematic reviews and meta-analyses: The PRISMA statement. Int J Surg.

[6] Shrutha SP, Vinit GB (2015). Rhabdomyosarcoma in a pediatric patient: A rare case report. Contemp Clin Dent.

[7] Peters SM, Kunkle T, Perrino MA, Philipone EM, Yoon AJ (2017). Mandibular embryonal rhabdomyosarcoma with cartilaginous metaplasia: report of a case and review of literature. Oral Surg Oral Med Oral Pathol Oral Radiol.

[8] Silva Cunha JL, De Assis Almeida Lima-Júnior F, Gonçalves Júnior WD, De Santana Santos T, Da Silveira EJD, De Sousa SF (2019). Embryonal Rhabdomyosarcoma (Botryoid Subtype) Affecting the Buccal Mucosa. Head Neck Pathol.

[9] Morales RL, Álvarez A, Esguerra J, Prada Avella MC, Rojas F (2019). Primary Conjuntival Rhabdomyosarcoma in a Pediatric Patient. Cureus.

[10] Beghdad M, Mkhatri A, Berrada O, Abada R, Mahtar M (2020). Embryonal mastoid rhabdomyosarcoma in a three years old child: A case report. Int J Surg Case Rep.

[11] Masoudi MS, Zafarshamspour S, Ghasemi-Rad M, Soleimani N, Lincoln C (2020). Intracranial Rhabdomyosarcoma of the Cerebellopontine Angle in a 6-year-old Child: A case report. J Pediatr Neurosci.

[12] Bhandarkar A, Menon A, Kudva R, Pujary K (2020). Embryonal Rhabdomyosarcoma-A Mimicker of Squamosal Otitis Media. Iran J Otorhinolaryngol.

[13] Kumar A, Pothula V (2021). Parameningeal alveolar rhabdomyosarcoma in a child. BMJ Case Rep.

[14] Karanian M, Pissaloux D, Gomez-Brouchet A, Chevenet C, La Loarer F, Fernandez C (2020). et al. SRF-FOXO1 and SRF-NCOA1 Fusion Genes Delineate a Distinctive Subset of Well-differentiated Rhabdomyosarcoma. Am J Surg Pathol.

[15] Fung CK, Chow C, Chan WK, Choi E, To KF, Chan J (2022). Spindle cell/sclerosing rhabdomyosarcoma with DCTN1::ALK fusion: broadening the molecular spectrum with potential therapeutic implications. Virchows Arch.

[16] Shahidatul-Adha M, Saizul Z, Hussain FA, Abdullah B (2021). Pediatric Sinonasal Rhbdomyosarcoma Presented as Squint: A Case Report and Literature Review. Cureus.

[17] Waldman LE, Williamson AK, Amodio JB, Collins L (2018). Congenital Rhabdomyosarcoma Presenting as a Neck Mass at Birth. Case Rep Pediatr.

[18] Iatrou I, Theologie-Lygidakis N, Schoinohoriti O, Tzermpos F, Vessala AM (2017). Rhabdomyosarcoma of the maxillofacial region in children and adolescents: Report of 9 cases and literature review. J Craniomaxillofac Surg.

[19] Reilly BK, Kim A, Peña MT, Dong TA, Rossi C, Murnick JG (2015). Rhabdomyosarcoma of the head and neck in children: Review and update. Int J Pediatr Otorhinolaryngol.

[20] El Demellawy D, McGowan-Jordan J, de Nanassy J, Chernetsova E, Nasr A (2017). Update on molecular findings in rhabdomyosarcoma. Pathology.

[21] Choi JH, Ro JY (2021). The WHO Classification of Tumors of Soft Tissue: Selected Changes and New Entities. Adv Anat Pathol.

[22] Rudzinski ER, Kelsey A, Vokuhl C, Linardic CM, Shipley J, Hettmer S (2021). Pathology of childhood rhabdomyosarcoma: A consensus opinion document from the Children's Oncology Group, European Paediatr Soft Tissues Sarcoma Study Group, and the Cooperative Weichteilsarkom Studiengruppe. Pediatr Blood Cancer.

[23] Anderson WJ, Doyle LA (2021). Updates from the 2020 World Health Organization Classification of Soft Tissue and Bone Tumours. Histopathology.

[24] Agaram NP, Chen CL, Zhang L, LaQuaglia MP, Wexler L, Antonescu CR (2014). Recurrent MYOD1 mutations in pediatric and adult sclerosing and spindle cell rhabdomyosarcomas: evidence for a common pathogenesis. Genes Chromosomes Cancer.

[25] Alaggio R, Zhang L, Sung YS, Huang SC, Chen CL, Bisogno G (2016). et al. A Molecular Study of Pediatric Spindle and Sclerosing Rhabdomyosarcoma: Identification of Novel and Recurrent VGLL-2-Related Fusions in Infantile Cases. Am J Surg Pathol.

[26] Mosquera JM, Sboner A, Zhang L, Kitabayashi N, Chen CL, Sung YS (2013). Recurrent NCOA2 gene rearrangements in congenital/infantile spindle cell rhabdomyosarcoma. Genes Chromosomes Cancer.

